# Cancer health disparities in racial/ethnic minorities in the United States

**DOI:** 10.1038/s41416-020-01038-6

**Published:** 2020-09-09

**Authors:** Valentina A. Zavala, Paige M. Bracci, John M. Carethers, Luis Carvajal-Carmona, Nicole B. Coggins, Marcia R. Cruz-Correa, Melissa Davis, Adam J. de Smith, Julie Dutil, Jane C. Figueiredo, Rena Fox, Kristi D. Graves, Scarlett Lin Gomez, Andrea Llera, Susan L. Neuhausen, Lisa Newman, Tung Nguyen, Julie R. Palmer, Nynikka R. Palmer, Eliseo J. Pérez-Stable, Sorbarikor Piawah, Erik J. Rodriquez, María Carolina Sanabria-Salas, Stephanie L. Schmit, Silvia J. Serrano-Gomez, Mariana C. Stern, Jeffrey Weitzel, Jun J. Yang, Jovanny Zabaleta, Elad Ziv, Laura Fejerman

**Affiliations:** 1 Division of General Internal Medicine, Department of Medicine, University of California, San Francisco, San Francisco, CA USA; 2 Department of Epidemiology and Biostatistics, University of California, San Francisco, San Francisco, CA USA; 3 Departments of Internal Medicine and Human Genetics, and Rogel Cancer Center, University of Michigan, Ann Arbor, MI USA; 4 University of California Davis Comprehensive Cancer Center and Department of Biochemistry and Molecular Medicine, School of Medicine, University of California Davis, Sacramento, CA USA; 5 Genome Center, University of California Davis, Davis, CA USA; 6 Department of Cancer Biology, University of Puerto Rico Comprehensive Cancer Center, San Juan, Puerto Rico; 7 Division of Breast Surgery, Department of Surgery, NewYork-Presbyterian/Weill Cornell Medical Center, New York, NY USA; 8 Center for Genetic Epidemiology, University of Southern California Keck School of Medicine, Los Angeles, CA USA; 9 Cancer Biology Division, Ponce Research Institute, Ponce Health Sciences University, Ponce, Puerto Rico; 10 Samuel Oschin Comprehensive Cancer Institute, Cedars-Sinai Medical Center, Los Angeles, CA USA; 11 Department of Oncology, Lombardi Comprehensive Cancer Center, Georgetown University, Washington, DC USA; 12 Helen Diller Family Comprehensive Cancer Center, University of California San Francisco, San Francisco, CA USA; 13 Laboratorio de Terapia Molecular y Celular, IIBBA, Fundación Instituto Leloir, CONICET, Buenos Aires, Argentina; 14 Department of Population Sciences, Beckman Research Institute of City of Hope, Duarte, CA USA; 15 Interdisciplinary Breast Program, New York-Presbyterian/Weill Cornell Medical Center, New York, NY USA; 16 Slone Epidemiology Center at Boston University, Boston, MA USA; 17 Department of Medicine, Zuckerberg San Francisco General Hospital and Trauma Center, University of California, San Francisco, San Francisco, CA USA; 18 Division of Intramural Research, National Heart, Lung and Blood Institute, National Institutes of Health, Bethesda, MD USA; 19 Office of the Director, National Institute on Minority Health and Health Disparities, National Institutes of Health, Bethesda, MD USA; 20 Division of Hematology/Oncology, Department of Medicine, University of California San Francisco, San Francisco, CA USA; 21 Subdirección de Investigaciones - Instituto Nacional de Cancerología de Colombia, Bogotá, Colombia; 22 Department of Cancer Epidemiology, H. Lee Moffitt Cancer Center and Research Institute, Tampa, FL USA; 23 Grupo de investigación en biología del cáncer, Instituto Nacional de Cancerología, Bogotá, Colombia; 24 Departments of Preventive Medicine and Urology, Keck School of Medicine of USC, Norris Comprehensive Cancer Center, University of Southern California, Los Angeles, CA USA; 25 Department of Medical Oncology and Therapeutics Research, City of Hope Comprehensive Cancer Center, Duarte, CA USA; 26 Department of Pharmaceutical Sciences, Department of Oncology, St. Jude Children’s Research Hospital, Memphis, TN USA; 27 Department of Pediatrics and Stanley S. Scott Cancer Center LSUHSC, New Orleans, LA USA

**Keywords:** Cancer epidemiology, Cancer epidemiology

## Abstract

There are well-established disparities in cancer incidence and outcomes by race/ethnicity that result from the interplay between structural, socioeconomic, socio-environmental, behavioural and biological factors. However, large research studies designed to investigate factors contributing to cancer aetiology and progression have mainly focused on populations of European origin. The limitations in clinicopathological and genetic data, as well as the reduced availability of biospecimens from diverse populations, contribute to the knowledge gap and have the potential to widen cancer health disparities. In this review, we summarise reported disparities and associated factors in the United States of America (USA) for the most common cancers (breast, prostate, lung and colon), and for a subset of other cancers that highlight the complexity of disparities (gastric, liver, pancreas and leukaemia). We focus on populations commonly identified and referred to as racial/ethnic minorities in the USA—African Americans/Blacks, American Indians and Alaska Natives, Asians, Native Hawaiians/other Pacific Islanders and Hispanics/Latinos. We conclude that even though substantial progress has been made in understanding the factors underlying cancer health disparities, marked inequities persist. Additional efforts are needed to include participants from diverse populations in the research of cancer aetiology, biology and treatment. Furthermore, to eliminate cancer health disparities, it will be necessary to facilitate access to, and utilisation of, health services to all individuals, and to address structural inequities, including racism, that disproportionally affect racial/ethnic minorities in the USA.

## Background

Considerable progress has been made over the past decade in describing cancer health disparities by racial/ethnic categories,^1–5^ as well as implementing changes regarding how racial/ethnic groups are defined. In the context of this review, we use the terms ‘race/ethnicity’ or ‘racial/ethnic minority populations’ to refer to what we know are heterogeneous groups of people defined by the USA Office of Management and Budget as African Americans/Blacks (AA/B), American Indians and Alaska Natives (AI/AN), Asians, Native Hawaiians/other Pacific Islanders and Hispanics/Latinos.^6^ We understand that these categories are socially constructed and relevant for the USA population based on their use within official registries, health systems and the decennial census.^7^ Because great diversity exists within broad racial/ethnic categories, studies from the past 15 years that report cancer incidence and outcomes often include subpopulation analyses by geography, country of origin, socioeconomic status (SES) or genetic ancestry.^2,8–22^

Research into health disparities has shifted from single-dimension models to complex frameworks that incorporate multiple domains (biological, behavioural, physical/built environment, sociocultural environment and healthcare system) and levels of influence (individual, interpersonal, community and societal).^6,23–27^ These ecosocial/multilevel frameworks attempt to provide a thought structure that integrates ‘biology’ and ‘social’ analyses to bring about new hypotheses instead of simply trying to combine or interpret associative results obtained within these different dimensions.^27^ However, progress in understanding the mechanisms (moving from association to causality) and subsequent reduction or elimination of disparities has been relatively slow. The slow rate of progress has been partly due to the lack of adequate support, data and biospecimen availability for research focused on diverse populations. Studies that combine biospecimen collection with measures of individual behaviour, physical/built and sociocultural/socioeconomic environment can be expensive due to the fact that these studies have to be large enough to be able to assess the independent and joint effects of these factors, and for distinct racial/ethnic groups. The development of large and prospective cohort studies, such as the Multiethnic Cohort (MEC) study (Box 1) has been rare, given the high costs associated with the infrastructure requirements for recruitment, baseline measures and long-term follow-up of a large number of participants from different race/ethnicities. These challenges, combined with limited immediate statistical power for subgroup analyses, add to the paucity of adequately diverse datasets for cancer research.^28–31^ Fortunately, current funding and resource development efforts, as well as ongoing international collaborations, aim to improve our knowledge of the basis of health/disease in diverse populations.^19,32–40^ Additional and continuous investments will be necessary to support studies that capture multidimensional and multilevel data, and allow a deeper understanding of how different factors interact to contribute to the observed cancer health disparities.

We begin our review by outlining factors that are commonly described as contributing to health disparities for multiple cancer types. Additional sections focus on the most common types of cancer (breast, prostate, colorectal and lung), and a selection of other cancer types that illustrate how different factors can contribute to disparities (gastric, liver, pancreas and leukaemia). Our overall aim is to provide a summary of some of the known cancer health disparities by race/ethnic categories in these selected cancer types, and the proposed contributing/associated factors. This is not an exhaustive review, and additional literature on health disparities for other cancer types, such as melanoma, bladder or ovarian cancer, can be found elsewhere.^41–43^ In the ’Conclusions‘ section, we describe suggested actions (Box 2) that could contribute to the elimination or reduction of cancer health disparities.

Box 1: The Multiethnic Cohort (MEC) study^366^

- The MEC is a large epidemiological study created in 1993–1996 and financed by the USA National Cancer Institute. It includes over 215,000 men and women primarily of five racial/ethnic groups: Non-Hispanic White, Japanese American, Native Hawaiian, African American and Hispanic/Latino participants.
- *Main goal:* MEC study aims to understand racial/ethnic differences in cancer as well as other chronic diseases, to contribute in correcting health disparities and prevent cancer and other diseases in all populations.
- *Resources***:** MEC collects baseline information through extensive questionnaires encompassing demographic, lifestyle, dietary, environmental and genetic risk factors, and performs follow-up questionnaires over time. Biological specimens (mainly blood and urine) have been collected from ~70,000 participants.

Box 2: Summary of suggested actions to advance research and reduce/eliminate cancer health disparities

- Further develop and sustain large diverse cohorts that collect multidimensional/multilevel data.
- Diversify germline and tumour genetics/genomics databases and clinical trials.
- Develop diverse cell lines and patient-derived xenograft models.
- Implement system changes in healthcare coverage to guarantee equity in access to high-quality screening and access to treatment.
- Improvement and system-wide implementation of patient navigation programmes.
- Employ culturally tailored community awareness and education programmes to increase cancer screening (including genetics) and modify risk behaviours.
- Implement legislation that supports behavioural interventions (e.g. limit the sales of tobacco products).

## Factors that contribute to health disparities for multiple types of cancer

Cancer health disparities affecting diverse populations in the USA are the consequence of the interplay of many factors. Some of them, such as those related to structural inequities, can lead to disparities observed in multiple types of cancer. This section provides a brief overview of some of those factors.

### Healthcare

The USA healthcare system is a combination of publicly and privately funded systems and programmes. The majority of Americans are covered by private insurance plans through their employers, and public programmes, such as Medicaid and Medicare, provide coverage to individuals with limited income and resources.^44^ However, even after the implementation of the Affordable Care Act in 2010, millions of Americans remain uninsured, with Hispanics/Latinos being the most affected group.^44,45^ In addition, few USA systems offer integrated care (e.g. the Kaiser Permanente model); under most health insurance companies or programmes, it is up to the patient to identify doctors and specialists and coordinate their own care. Racial/ethnic minorities are known to be disproportionally affected by this approach,^44^ due in part to cultural and language barriers.^46^

### Socioeconomic status

Self-reported race/ethnicity correlates with SES,^47^ and, in turn, low SES has been associated with poor access to high-quality care, lower screening rates, delays in treatment after diagnosis and lower treatment adherence.^48^ Cancer health disparities between racial/ethnic minorities and Non-Hispanic Whites (NHWs) can be in part explained by these associations.^49–53^ Furthermore, financial distress associated with cancer management prevents adequate care, starting prior to diagnosis, during imaging and through pathological confirmation.^54,55^ Whereas effective advances in multidisciplinary cancer care have contributed to improved survival rates, the costs of these treatments and the concomitant impact of some treatments on employment/disability disproportionately burden those patients who are socioeconomically disadvantaged, many of whom are AA/Bs and Hispanics/Latinos.^54,56,57^ However, SES is not the only dimension to consider when thinking about disparities affecting racial/ethnic minorities—additional factors are associated with SES but not fully encompassed by it. For example, among immigrant populations who have not long been in the USA, limited English proficiency can be an additional barrier to accessing healthcare services.^58–63^

### Other risk factors (modifiable and non-modifiable)

The relatively high prevalence of certain underappreciated exposures might also contribute to a higher incidence of different types of cancer. For example, AA/Bs and Hispanics/Latinos in the USA are more likely to live in low-income areas that are exposed to higher levels of environmental pollution.^64–69^ Independent of SES, individuals from racial/ethnic minorities might be exposed to socio-environmental conditions and stressors that affect health outcomes throughout the course of life. For example, structural and interpersonal racism can translate to a higher risk of psychosocial stressors.^47^

In addition, disparities observed for some types of cancer are also explained by differential incidences of infections or diseases by race/ethnicity, such as cytomegalovirus (CMV) infection for childhood acute lymphoblastic leukaemia,^70,71^ and hepatitis B (HBV) and HCV for liver cancer.^72,73^

Previous studies point to further stratification of cancer incidence and mortality in diverse populations based on birthplace. Some studies on breast cancer^15,16^ and prostate cancer^15,74–76^ have shown that incidence and mortality rates are different among different Latin American countries. Differences have also been observed for different populations of AA/Bs.^77^ Some cancer types show a higher rate of cancer-specific mortality in second- and third-generation immigrants compared with their foreign-born counterparts.^10,77–81^ These findings suggest that the adoption of certain behaviours (e.g. changes in diet or reproductive behaviour) or environmental exposures that are more prevalent in the USA might lead to an increased risk of mortality for certain cancers.^82–95^ Because the country of origin is missing for a large proportion of cancer patients in the Surveillance, Epidemiology and End Results (SEER) Program data, descriptive analyses on incidence and mortality by country of origin based on this resource must be interpreted with caution.^96^

Substantial evidence has shown that unequal cancer burden among populations of different races/ethnicities can be partially explained by their population-specific genetic background or genetic ancestry.^49,97,98^ For example, Hispanics/Latinos are part of a genetically diverse ethnic group with varying proportions of Indigenous American, European, African and to a lesser extent, Asian ancestry components.^99^ Inclusion of genetic ancestry in studies focused on the molecular biology of cancer in admixed populations is essential to prevent confounding.^100^ Genetic ancestry in admixed populations can also be leveraged to identify ancestry-specific biology,^101,102^ and provide insights into observations such as the disproportionally higher incidence of triple-negative breast cancer (TNBC) and aggressive prostate cancer in AA/Bs, or lower incidence of breast cancer in Hispanics/Latinas.^102–105^

Race/ethnicity and SES are highly correlated, and often they are studied separately.^106^ After adjusting for SES, disparities in cancer risk and outcomes are reduced but not eliminated.^106,107^ Intersectional approaches that focus on the complex interaction of social determinants with other factors that are experienced simultaneously, can provide an opportunity to disentangle their joint effect on the observed disparities.^108^

## Disparities in breast cancer

### Incidence and mortality

Age-adjusted breast cancer incidence varies by race/ethnic category, being the highest in White individuals (131.3/100,000), followed by AA/Bs (124.8), Asians and Pacific Islanders (102.9), Hispanics/Latinos (99.1) and AI/ANs (79.5).^109^ It is notable that although breast cancer incidence has historically been relatively low among Asian groups,^110^ it has been increasing over the past 30 years.^57,110^ The few reports that have disaggregated the incidence and mortality of breast cancer among Asian subgroups have shown strikingly variable rates and trends.^13,110,111^ Similar variation has been observed for different Hispanic/Latino populations.^15^

AA/B, AI/AN and Hispanic/Latina women have a higher risk of breast cancer-specific mortality relative to NHW women.^112–117^ This increased risk is consistent with studies describing a more advanced stage at diagnosis,^21,112^ lower treatment adherence,^118^ limited access to high-quality care^119^ and a higher risk of developing the most aggressive subtypes of breast cancer among individuals from racial/ethnic minorities compared with NHWs.^114,120–124^

### Disease subtypes

AA/B women of West African descent and Hispanics/Latinas in the USA and abroad are more likely to be diagnosed with hormone-receptor-negative (HR^–^) tumour phenotypes than patients from other populations.^56,57,121–123,125,126^ Hispanics/Latinas and Asian women are also at increased risk of being diagnosed with human epidermal growth factor receptor 2-positive (HER2^+^) disease.^18,19,121–123,125^ AA/Bs are twice as likely as NHW women to have TNBC (indicating the lack of oestrogen receptor [ER], progesterone receptor [PR] and HER2).^19^ TNBC is one of the most aggressive subtypes of breast cancer, with as yet no targeted treatment. The higher incidence of HR^–^ breast cancer in AA/B women and Hispanics/Latinas might be due to different levels of exposure to environmental and lifestyle factors that play a role in the aetiology of this type of breast cancer. Evidence shows that higher parity in the absence of breastfeeding is associated with an increased risk of HR^–^ breast cancer,^93^ and in the USA, breastfeeding prevalence is markedly lower in AA/B women than in other populations.^93,94^ Furthermore, type 2 diabetes and obesity, which are more common in AA/B and Hispanics/Latinos than NHWs,^127^ can also increase the risk and progression of breast cancer, particularly HR^–^ breast cancer.^84,91,92^ Insulin resistance, a risk factor for prediabetes and type 2 diabetes, promotes weight gain and in turn induces tissue inflammation.^128^ Inflammatory cytokines and associated immune cells involved in this process activate signalling pathways that promote more aggressive TNBC phenotypes.^128^ In addition, AA/Bs and Hispanics/Latinas are more likely to live in low-income areas with higher exposures to environmental pollution, and emerging epidemiology evidence supports a possible role for hazardous air pollutants, traffic emissions and radon in breast cancer, particularly HR^–^ breast cancer.^64,65^

The specific factors contributing to the higher prevalence of HER2^+^ breast cancer in Asians and Hispanics/Latinas are unknown. However, a study reporting a positive association between the proportion of Indigenous American ancestry and HER2 status in breast cancer patients from Peru, Colombia and Mexico suggested that germline genetic variants associated with this component of ancestry might play a role.^129^ It is also possible that other, as yet unknown, factors that are highly correlated with ancestry proportions in these populations can explain the observed association.^129^

### Genetic variants

Genome-wide association studies (GWAS) have discovered several loci associated with an increased risk of breast cancer;^130–135^ however, these variants have been primarily described in European populations, while other populations remain underrepresented.^29^ GWAS hits have been replicated in Hispanic/Latino populations but poorly in AA/B populations, probably due to distinct linkage-disequilibrium patterns.^136^ The predisposition to breast cancer by common genetic variants differs according to genetic ancestry,^105,137^ and ongoing efforts are aimed at detecting population-specific risk variants for breast cancer in AA/B,^138–140^ Asian^141,142^ and Hispanic/Latina^105,138^ women. The presence of a single-nucleotide polymorphism (SNP) at 6q25 among Hispanics/Latinas is associated with a lower risk of breast cancer, especially HR^–^ subtypes.^8,101,143^ In addition, risk variants that are more common in women of African genetic ancestry have been reported to be associated with HR^–^ disease.^139,140^ One of these SNPs is located within *TERT* (telomerase reverse transcriptase),^139^ a known cancer-susceptibility gene. However, the molecular mechanisms underlying the observed associations are not yet clear.

GWAS-identified SNPs with small individual effects can be combined into polygenic risk scores (PRS) to predict cancer risk. Given the limited availability of racial/ethnic diverse samples, PRS generated with SNPs discovered mostly in European or Asian populations are being tested for their predictive power among individuals from other groups.^37,144,145^ The results suggest that, as currently calculated, these PRS are less predictive of cancer risk in individuals with high African ancestry,^144,145^ but are equally predictive among Hispanics/Latinas, even in those of high Indigenous American ancestry.^37^ SNPs discovered in European populations can, therefore, be used to predict the risk of breast cancer in non-Caribbean Hispanics/Latinas, thus widening the application of PRS.

Regarding high-penetrance mutations associated with an increased risk of breast cancer, Hispanics/Latinas from certain regions might have higher rates or a different set of mutations compared with NHWs.^146–150^ Although the frequency of *BRCA* mutations in AA/Bs is lower than the frequency in Hispanics/Latinos and non-Ashkenazi Jewish NHWs, AA/Bs have higher rates of variants of unknown significance (VUS),^151^ comparable with the frequency observed among Asians.^152,153^ Over time (within months to years of their initial classification), as more information is gained about normal human genomic diversity, most (90.3%) VUS are downgraded to benign/likely benign variants; only 7.5% of the VUS are reclassified as pathogenic/likely pathogenic.^154^ This evidence highlights the need for additional genetic/genomic studies to understand the significance of unclassified variants in diverse ancestry groups.

### Tumour biology

Differences in tumour biology according to race/ethnicity have also been described. Studies have found differences in gene expression^155–157^ and methylation patterns^158–161^ between AA/Bs and NHWs, which might have a potential impact on patient outcomes.^156–160,162^ Most of these findings are more evident for young, HR^–^ patients.^155,158,161^ Other features, such as a differential mutational landscape^157,163^ and higher frequency of DNA copy number alterations,^161^ have been reported for AA/Bs compared with NHWs, in addition to the existence of differential immune and inflammatory pathways involved in tumour-specific immune responses between the two groups.^164,165^ As stated above, obesity is associated with increased circulating levels of insulin and inflammatory cytokines, such as IL-6, IL-8 and TNF-α and CD8^+^ T cells and M1 macrophages, which contribute to the development of a pro-tumorigenic microenvironment and more aggressive tumour characteristics, leading to TNBC biology in AA/B women.^128,166^ Breast cancer research that is focused on the interplay between race/ethnicity, poverty, diet, obesity and aggressive tumour biology exemplifies the richness and complexity of hypotheses that result from the use of ecosocial/multilevel theoretical frameworks.

Few studies have evaluated differences in tumour biology among Asians or Hispanics/Latinas compared with NHWs.^167–169^ One study, based on the TCGA database, described the differential activation of several cancer-related pathways between Asian Americans and NHWs.^167^ Another study compared a Korean breast cancer cohort with NHWs from the TCGA, and found differences in mutational profiles and other differences driven by features associated with the tumour microenvironment, leading to a more immune-active tumour microenvironment among Asians.^168^ A small study using Oncotype DX^169^ suggested that *CCNB1* and *AURKA* genes are highly expressed in Hispanics/Latinas, and that Hispanics/Latinas with early-stage HR^+^/HER2^–^ tumours have increased proliferation compared with NHWs.^169^ For other populations, more studies are needed to address disparities, with a focus on tumour biology beyond genetics.

## Disparities in prostate cancer

### Incidence and mortality

Prostate cancer is the number 1 cancer affecting men in the USA, accounting for 20% of all cancers, and the second highest cause of cancer-related deaths, with 33,330 men predicted to die of this disease in 2020.^2^ In more than 80% of men diagnosed with prostate cancer, the disease will be localised, indolent and, if left undetected, would be harmless. However, a significant subset of prostate tumours will be aggressive and can lead to death. Interestingly, prostate cancer incidence rates have declined over the past 10 years, which might be due in part to the US Preventive Services Task Force (USPSTF) recommendation^170^ in 2012 against routine screening because of concerns of overdiagnosis and overtreatment, as well as other undefined factors.^171^ A shift towards increases in metastatic and lethal prostate cancer appears to be occurring,^172^ especially among younger AA/B men.^173^ Due to early detection and treatment advances, mortality from prostate cancer has decreased over the past two decades by 51%,^48^ but, unfortunately, this improvement has not benefited all equally, as racial and ethnic disparities persist.

Prostate cancer disparities constitute the largest of all cancer disparities. AA/B men suffer disproportionately from prostate cancer, facing a 78% higher incidence rate than NHW men.^2^ AA/B men are also more likely to be diagnosed at a younger age, present with more advanced and aggressive disease and have a 2.3-times higher mortality rate compared with NHW men.^2,174,175^ Although the incidence rates for prostate cancer are lower in Hispanics/Latinos and some Asian groups than in NHW men,^174^ Hispanics/Latinos are more likely to present with more advanced-stage disease.^176^

The incidence of prostate cancer also varies among different racial/ethnic populations based on the place of residence or country of origin. For example, among Hispanics/Latinos, the incidence is lower for Mexican Latinos than Caribbean Latinos.^15,74–76^ Inhabitants of Puerto Rico have lower incidence rates than Puerto Ricans living in mainland USA.^177^ Hawaiians/Samoans living in Los Angeles have incidence rates of prostate cancer higher than NHWs living in Los Angeles, followed by Filipinos and Japanese, who have a lower incidence than NHWs but a higher risk than other Asian groups.^1^ Although patients from some Asian subpopulations have better survival rates than NHW patients, they are more likely to present with advanced disease and metastatic prostate cancer, particularly those who are foreign-born.^48,178,179^ This contradictory behaviour is yet not well understood; however, the authors speculate that this might be attributable to biological and lifestyle factors.^179^

### Potential aetiological factors

Few established risk factors exist for prostate cancer. Among them are non-modifiable risk factors such as age, African genetic ancestry, family history of prostate cancer and common genetic variants.^180–182^ AA/B men not only have a higher risk of developing prostate cancer, but they tend to have a more aggressive disease. Several genetic variants at the 8q24 locus^183,184^ and other loci^185^ are more common in AA/B men, and might explain some of the differences in incidence and outcome between AA/Bs and NHWs. In addition, a mutation in a prostate cancer tumour-suppressor gene (*EphB2*) was reported to have a higher frequency among AA/B men, which may also explain the role of family history and African ancestry in increasing the risk of prostate cancer.^186^ Differences in microRNA regulation may also contribute to exacerbate the observed increased aggressiveness of tumours among AA/B men.^187^ Furthermore, tumour gene mutations that are common among NHWs, such as *PTEN* and *TMPRSS2–ERG* fusions, might be less prevalent among AA/Bs, and novel mutations have been reported in genes not previously thought to play a key role in prostate cancer, such as *CDC27–OAT* fusion and *ERF*.^188–190^ The most recent study with tumour and follow-up data suggests that copy number alterations, TP53 somatic mutations and deletions in CDKN1B may be associated with poor outcome among AA/B men.^191^ The tumour mutational landscape of AA/B and Hispanic/Latino prostate cancer patients needs to be better characterised. Altogether, fewer than ~250 prostate tumours from AA/Bs have been studied, and to our knowledge, no tumours from Hispanics/Latinos are included in existing characterisation studies.^31^

Other probable modifiable risk factors include calcium, vitamin D and lycopene intake,^192^ body fatness^193^ and red meat intake,^194^ which are known to vary by racial/ethnic groups.^195,196^ Among Hispanics/Latinos, exposure to agrichemicals has also been reported as a risk factor.^197,198^

### Healthcare: screening and treatment

The excess burden of prostate cancer borne by AA/B men should be an urgent public health priority. Men of African descent have essentially been unrepresented in prostate-specific antigen (PSA) screening trials, but rigorous modelling studies carried out in 2017 have shown that PSA screening can yield greater mortality benefits for high-risk groups, especially AA/B men.^199^ Early detection remains paramount, and individuals can benefit from PSA-based screening as currently recommended by the USPSTF.^200^ A high midlife (mid-to-late 40s) baseline PSA test strongly predicts the likelihood of developing lethal prostate cancer in the future, particularly among AA/B men, and conversely, a low value rarely leads to aggressive prostate cancer in the future, and can therefore minimise the need for screening in low-risk men.^201,202^ However, early detection alone will not eliminate the disparities in prostate cancer. How the complex interplay between social factors (e.g. racism) and biology (e.g. genomic differences) contributes to prostate cancer disparities has not been clearly elucidated. A need for multilevel data exists, as does the need to develop approaches to identify risk factors and reduce them, by fully engaging all stakeholders, including patients, providers, community members and organisations and healthcare systems.^203^

Access to, and utilisation of, healthcare is a key factor in racial/ethnic disparities. For example, the reported overall lower incidence among AI/AN and Hispanics/Latinos might be explained by lower PSA screening rates compared with NHW men,^204–206^ raising concerns about under-detection in these populations. This lack of early detection contributes to higher mortality, particularly among AI/AN men.^207^ Standard prostate biopsies are key to diagnosis and cancer staging.^208^ However, this approach is limited in its ability to accurately visualise and target prostate lesions, and is thereby prone to under-sampling and under-diagnosis of clinically significant prostate cancer. Multiparametric MRI-guided biopsy sampling can address these challenges,^209^ but this procedure is less likely to be available to low SES patients.^210^ The rate of under-diagnosis seems to be the highest for AA/Bs, followed by Hispanics/Latinos and then NHWs.^211–213^

Individuals from racial/ethnic minority populations and uninsured patients are more likely to experience delays in treatment than insured, NHW individuals,^48,53^ and the treatment for AA/B men with high-risk/aggressive prostate cancer is less likely to be definitive (involving surgery or radiation); these disparities are the greatest in low-income communities.^214^ Furthermore, men from racial/ethnic minority populations and those on a lower income reported worse bowel and urinary function and more sexual dysfunction than NHW men after radical prostatectomy or radiation, which might reflect poorer-quality treatment and follow-up care, as well as the disadvantages prior to treatment.^215^ Given that most cases of prostate cancer are localised, adequate risk stratification at the time of biopsy is critical to avoid treatments that impact the quality of life, and to ensure that aggressive tumours receive definitive treatment. Unfortunately, current risk stratification biomarkers and risk models do not adequately represent diverse populations.^103^

## Disparities in lung cancer

### Incidence and mortality

Lung cancer is the leading cause of cancer mortality in the USA; however, the incidence and mortality rates for lung cancer vary substantially by self-reported race and ethnicity. AA/B men have the highest incidence rate (71.2/100,000 people) compared with other racial/ethnic groups (35.1–65.3).^216^ Among women, the lowest incidence rates have been observed in Hispanics/Latinas and Asians (24.8 and 28.6 out of 100,000, respectively), which are about half of those for NHW and AA/B women.^216^

### Smoking and lung cancer incidence

On the basis that tobacco smoking causes 80–90% of all cases of lung cancer in the USA,^217^ smoking rates should be a robust predictor of incidence. However, analyses from the MEC study of 1979 cases of lung cancer showed that for a similar smoking history of up to 30 cigarettes per day (CPD), AA/Bs and Native Hawaiians had a significantly higher relative risk (RR) of lung cancer compared with NHWs (RR = 0.57 for 11–20 CPD), Hispanics/Latinos (RR = 0.36 for 11–20 CPD) and Japanese Americans (RR = 0.39 for 11–20 CPD).^218^ A follow-up analysis of the MEC study with 4993 cases confirmed the higher rates for AA/Bs and Native Hawaiians and lower rates for Hispanics/Latinos.^219^ When modelling RR from exposure to 50 pack-years, after adjustment for total nicotine equivalents, the excess risk for AA/Bs was accounted for, as were the lower risks for Japanese and NHWs.^219^ However, the higher risk for Native Hawaiians and the lower risk for Hispanics/Latinos remained unexplained.^219^ Thus, given similar exposure to the same carcinogen, the rate of lung cancer differed by a factor of 2–3 according to self-reported race/ethnicity.

**Fig. 1:**
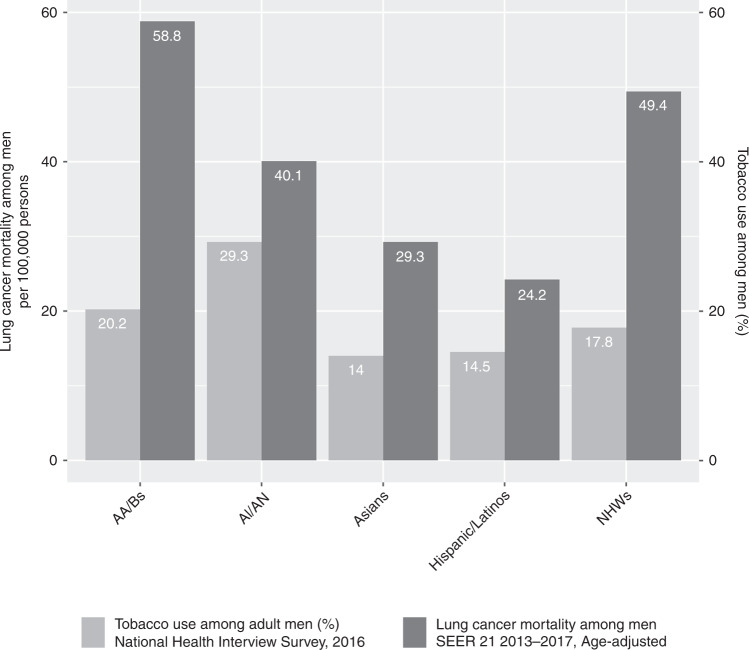
Lung cancer mortality rates and tobacco use among adult men in the United States by racial/ethnic category. Bars in light gray represent age-adjusted lung cancer mortality rates in the USA for the period 2013–2017^216^ and bars in dark gray represent tobacco use among adult men as reported by the 2016 National Health Interview Survey,^365^ stratified by racial/ethnic category.

The optimal strategy for preventing lung cancer is tobacco control. Some populations have benefitted from the steady decline of smoking rates in adults over the past 50 years. In national surveys, Hispanic/Latina women (7%) and women from some Asian populations (4.6%), and Hispanic/Latino men (14.5%) and men from some Asian populations (14%), have lower prevalence of smoking than NHW men (17.8%).^220^ Variation by national background occurs with a higher prevalence for Chinese immigrant men and Puerto Rican mainland women.^221,222^ AA/B men are more likely to smoke (20.2%) than NHW men, whereas smoking prevalence is lower among AA/B women compared with NHW women (13.5% vs. 15.5%).^220^ Smoking prevalence is the highest among AI/ANs (29.3% for men and 34.3% for women), and recent surveys show similar proportions for persons identified as being ‘more than one race’.^220^ Smoking therefore correlates with the observed incidence of lung cancer, except for the disproportionate excess risk among AA/B and the lower risk among Hispanics/Latinos (Fig. 1).^218^

Other aspects of smoking behaviour might partially explain the differences in lung cancer incidence: very light (1–5 cigarettes per day) daily smokers or non-daily smoking among Hispanics/Latinos,^222^ smoking topography (puff volume, duration, flow, etc.), menthol cigarette smoking among AA/B and interactions between environmental factors and smoking,^223^ novel alleles in chromosomes 2 and 4 associated with non-daily smoking among Hispanics/Latinos,^87^ less successful cessation and higher dependence among AA/B^88^ and low uptake of pharmacological cessation aids among individuals from racial/ethnic minority groups.^88^ Additional postulated factors include undefined genetic and epigenetic mechanisms, as well as second-hand smoke, which is most common among AA/Bs and persons living below the poverty line.^224^

### Healthcare: screening and treatment

One way to reduce lung cancer mortality, by about 20%, is through the use of low-dose computed tomography screening followed by a diagnostic evaluation if the result was abnormal.^225^ However, the uptake of such screening has been ≤6%,^226^ despite it constituting a prevention service for all insured persons. Only 9% of participants in the original trial were from a diverse racial/ethnic group, and the smoking history eligibility criteria might have limited the recruitment of diverse, lighter-smoking individuals.^225^ Concerns about morbidity owing to the need for bronchoscopy and biopsy as part of the screening might have also decreased the uptake, despite endorsement by clinicians.^227^ Research into screening strategies beyond computed tomography-led approaches is required, given the existing debate over the benefits and harms of this technique,^228^ and the potential for reducing disparities using life-gained-based eligibility criteria, which include younger current smokers with fewer comorbidities. This strategy has been shown to increase life expectancy per prevented lung cancer death (21.7 vs. 8.9 years, compared with risk-based strategy alone) and reduce potential screening-associated harms.^229^

AA/Bs are diagnosed at a younger age and with more advanced disease than NHWs. Although Hispanics/Latinos, especially those foreign-born, show lower mortality rates than NHWs, they are also diagnosed with more advanced-stage diseases.^230^ Both groups are less likely to receive standard of care,^230,231^ and to undergo guideline-recommended PET/CT imaging at diagnosis of non-small-cell lung cancer at all stages,^232^ and AA/Bs are less likely to be treated with immunotherapy-type compounds than NHWs, regardless of insurance status.^233^ These differences might contribute to the racial differences observed in survival. The survival rates for AA/Bs are lower than for NHW (16 and 19%, respectively for all stages).^174^ Nevertheless, under conditions of equal access to treatment, no differences in survival prevail,^234^ reinforcing the need of improving access to quality care for this population. The survival differences between AA/B and NHW patients with Medicare diagnosed with early-stage lung cancer are in part accounted for by a lower rate of surgical treatment (64% vs. 76.7%) according to SEER Program data.^235^ Surgical therapy has improved so that early-stage 3 non-small-cell lung cancer is often amenable to resection, and the persistent lower rate of surgery for AA/B patients might be amenable to system interventions.^236^ Limited data exist on attitudes towards treatment, stage at presentation and outcomes of therapy in other diverse populations with lung cancer.

Lung cancer among never smokers is becoming an increasing problem in populations with low rates of tobacco use, with over 50% of lung cancer cases being diagnosed in never-smoker East Asian women.^237^ East Asians are far more likely to be diagnosed with lung cancer that harbours somatic mutations in the epidermal growth factor receptor (*EGFR)* gene;^238,239^ this has important implications for treatment as these cancers are more likely to respond to tyrosine kinase inhibitors^240^ and prolong life expectancy by 2 years or more.^241^ Analyses of Medicare data from 2010 to 2013 showed a relative increase of up to 20% in testing of these mutations using tissue from lung cancer patients.^242^ Asians, women and never smokers were more likely to be tested, and AA/B, Hispanics/Latinos and patients on Medicaid were less likely to be tested.^242,243^

### Genetics and tumour biology

Comparative analyses of the molecular features of lung cancer tissue revealed racial/ethnic differences in genomic profiles, indicating that the somatic differences observed have genetic ancestry origins. AA/B patients present higher genomic instability, more aggressive molecular features^244^ and higher frequency of mutations in *PTPRT* and *JAK2* genes compared with NHWs.^245^ Asians and Hispanics/Latinos also presented different frequencies of mutations in driver genes compared with NHWs.^246,247^ Among Asians, the higher prevalence of mutations in *STK11*, *TP53* and *EGFR* genes could explain the better efficacy of PD‐L1 inhibitors in this population.^247^ Generating additional data on the frequency of these mutations by race, ethnicity and genetic ancestry will advance our understanding of the mechanisms of lung cancer. In addition, enhancing access to clinical testing for individuals from diverse populations will be critical for improving the quality of lung cancer care for all.

## Disparities in colorectal cancer

### Incidence and mortality

Substantial disparities in the incidence and mortality rates for colorectal cancer (CRC) exist in the USA among racially and ethnically diverse populations. AA/Bs have the highest incidence rates of CRC (45.7/100,000) as compared with AI/ANs (43.3), NHWs (38.6), Hispanics/Latinos (34.1) and some Asian populations (30.0).^2^ CRC mortality rates follow the same pattern, with the highest rates observed among AA/Bs (19.0/100,000), followed by AI/ANs (15.8), NHWs (13.8), Hispanics/Latinos (11.1) and some Asian subpopulations (9.5).^2^ The underlying factors driving disparities in CRC mortality have not been conclusively determined, although lower screening rates, more advanced stage at diagnosis, differences in treatment patterns and unique tumour biology are all believed to contribute.^76,248–252^

Aggregating heterogeneous populations, however, masks the significant variability that exists in both CRC incidence and mortality within subgroups. For example, Alaskan Natives experience the highest CRC incidence and mortality rates among all populations, and Cubans and Puerto Ricans have disproportionately higher rates of incidence and mortality than Hispanics/Latinos from other backgrounds.^16,20–22^ The mortality:incidence ratio demonstrates additional disparities that reflect treatment and survival differences in CRC patients.^253^ Indeed, the disease in AA/B and Hispanic/Latino CRC patients is less likely to be localised and regional, and therefore less amenable to the chance of cure by surgery or radiation, and more likely to be metastatic, compared with the disease in NHWs.^2,254^

### Potential aetiological factors

Proposed contributors to the development of CRC include individual genetic make-up as well as the macro- and microenvironment that might influence biological behaviour in the colon.^249,255^ The distribution of other risk factors for sporadic CRC might differ between groups, including high-fat/high- caloric diets, excess body mass index, low physical activity, usage of tobacco products, alcohol intake, low serum calcium and vitamin D levels and low fish oil intake.^82,83,256^ In addition, established protective factors, such as hormone replacement therapy in women and the use of aspirin and NSAIDs, are likely to be different between groups.^253,256^ Gene–environment interactions have also been shown to influence the risk of CRC—for example, the intake of red meat, a known risk factor for CRC, can interact with genetic variants in some of the key metabolism genes that are relevant for carcinogen activation and detoxification.^257,258^ However, very little is known about gene–environment interactions in diverse populations and how, or if, psychosocial, behavioural or other exogenous agents might affect these interactions.^259^

### Genetics and tumour biology

The extent to which germline genetics and tumour biology contribute to racial/ethnic disparities in CRC outcomes has received limited attention. A few studies to date have investigated and identified differential responses to cancer therapy and CRC-specific survival across populations. For example, findings from a clinical trial of stage III CRC patients suggested that racial/ethnic disparities in survival persist despite uniform treatment.^260^ NHWs experienced better response and greater toxicity from fluorouracil (FU)-based adjuvant therapy regimens than did AA/Bs, with differences in the frequency of pharmacogenetic variants across the populations cited as likely contributors.^261,262^ Differences in the prevalence of microsatellite instability/mismatch-repair deficiency, an important molecular feature of some CRCs, might also affect the response to FU-based therapies.^263^ Furthermore, a survival benefit has been observed for chemotherapy and biologic agents versus chemotherapy alone for NHWs but not for AA/B or Hispanics/Latino patients.^264^ Finally, novel somatic mutations (in *HTR1F, FLCN* and *EPHA6*) identified exclusively in AA/B patients with CRC have been associated with adverse clinical outcomes, although these results require verification in other cohorts.^265^ No published data exist specifically for unique driver mutations among Hispanics/Latinos, AI/ANs, Asians or Hawaiian/Pacific Islanders. Further studies assessing the interaction between social (e.g. diet) and biological factors (e.g. gut microbiome, tumour and immune microenvironment and germline genetics) influencing CRC incidence and outcome disparities are warranted.

### CRC prevention

A key approach to stopping the development of CRC has been the implementation of prevention strategies, such as those promoting a healthy lifestyle or medication compliance.^266^ However, low SES and restricted healthcare access can limit proven approaches to prevention and, thus, strategies have not been uniformly applied across population groups.^56,253,267^ Interventions such as patient navigation could overcome these limitations: there is strong evidence that patient navigation in conjunction with the use of screening via faecal immunohistochemical tests and/or colonoscopy can eliminate disparities in the incidence of CRC.^268^

## Disparities in pancreatic cancer

### Incidence and mortality

Pancreatic cancer is uncommon but deadly, with almost 57,600 new cases and 47,050 deaths expected in 2020 in the USA.^2^ Among the few cancers for which the incidence has been steadily increasing (1–2% annual increase for more than a decade), pancreatic cancer also maintains one of the lowest overall 5-year survival rates of 9% (3% in more than 50% of patients diagnosed with metastatic disease).^2^ The lack of sensitive and specific early detection methods and limited treatment options (only 20% are eligible to undergo ‘curative’ surgical resection) have contributed to this poor prognosis. Long-standing and incompletely explained differences in the rates of pancreatic cancer incidence and mortality according to race/ethnicity have been documented since the 1970s.^174^ The historically low recruitment of minorities in pancreatic cancer research studies must be remedied to increase understanding and guide efforts to address these disparities.

AA/Bs have both a higher incidence^269–273^ and mortality rate^270,271,273–275^ compared with NHWs as well as other populations, which has been documented for both younger and older adults (<50 years vs. >50 years) across all USA states other than Hawaii.^174,271,272^ Nearly all racial/ethnic groups showed a steady increase in incidence over a 27-year period from 1988 to 2015, with AI/ANs (who had the lowest incidence of all race/ethnicity groups) showing the greatest increase over time and AA/Bs showing stable incidence rates.^272^ A small study of diverse individuals in New Mexico suggested that patients who self-identified as ‘Native American’ (most likely Navajo) had a higher mortality risk within 30 days of diagnosis, poorer 5-year survival and were less likely to receive chemotherapy than NHWs and Hispanics/Latinos.^276^

### Potential aetiological factors

Behavioural and lifestyle factors that are related to pancreatic cancer risk and prognosis, including smoking, diabetes, obesity and alcohol consumption, have been suggested to play a role in the observed disparities in incidence and mortality, especially among AA/Bs.^89,270^ In particular, sudden- onset diabetes has been reported to increase the risk of pancreatic cancer among AA/Bs and Latinos.^277^

The study of genetic factors that might contribute to racial/ethnic disparities in pancreatic cancer has been limited. There is some evidence of a higher prevalence of germline and somatic mutations in the genes encoding cyclin-dependent kinase inhibitor 2A *(CDKN2A)* and KRAS Proto-Oncogene, GTPase (*KRAS)*, in AA/Bs compared with NHWs.^278,279^ In addition, the risk genotypes of the P335L and P109S variants of the somatostatin receptor 5 (*SSTR5)* gene occur more frequently in AA/Bs than in NHWs or Hispanics/Latinos, and are also associated with reduced survival.^280^ As is the case for other types of cancer, an exploration of how biological and social factors interact to contribute to racial/ethnic disparities in pancreatic cancer is lacking and needed.

### Healthcare and treatment

Differences in diagnosis and treatment have been shown to contribute to some of the observed disparities in survival by race/ethnicity, especially for AA/Bs.^89,270,281,282^ Factors such as older age, minority race/ethnicity, lower SES, being uninsured or on Medicaid, higher comorbidity index and treatment at a non-academic centre or a low-volume hospital have been inversely correlated with receiving standard therapy including surgery,^281,283–288^ and are also associated with patient refusal of treatment.^275,289^

A study conducted within the Kaiser Southern California patient population (an integrated system), reported no racial/ethnic differences with regard to pancreatic cancer treatment and outcomes.^269^ This suggests that providing access to high-quality care to all individuals could eliminate the observed racial/ethnic disparities in pancreatic cancer survival.

## Disparities in gastric cancer

### Incidence and mortality

Each year, gastric cancer accounts for ~1 million new cancer cases and ~730,000 cancer deaths worldwide, representing the third biggest global cause of cancer mortality.^290,291^ Even though gastric cancer is relatively rare in NHWs (incidence rates for men of 7.6/100,000 and for women of 3.5/100,000),^2^ it remains a significantly disproportionate burden of disease in other populations, specifically in AA/Bs, Asians, Hawaiian/Pacific Islanders and Hispanics/Latinos. Data from the SEER Program show that individuals from these groups are ~1.5–2.0 times more likely than NHWs to be diagnosed with and die from gastric cancer.^290,292–296^ Among Asians in California, the highest rates have been reported among Japanese Americans and Koreans, with the latter showing double the rate than Japanese Americans and having the highest incidence rates in the USA.^297^

The high gastric cancer mortality rate highlights its poor prognosis. Although early-stage tumours are treatable, the vast majority of gastric cancers are diagnosed at advanced stages due to a lack of symptoms and limited early detection capability.^290,298–300^ The 5-year survival rate for metastatic gastric cancer is 5% in the USA.^292^ The incidence of gastric cancer has decreased over the past decades due to increased *Helicobacter pylori* screening and treatment, together with improvements in sanitation, hygiene, clean water and food preservation.^293^

Hispanics/Latinos, Asians and Hawaiian/Pacific Islanders and AI/ANs on average are diagnosed at an earlier age than NHWs, which could reflect the difference in the age distribution of these populations as well as earlier-onset disease.^294–296^ Hispanics/Latinos and Asian Americans, which together account for ~25% of the total US population,^301^ represent the largest and fastest-growing US minority populations, respectively.^302,303^ Notably, Hispanics/Latinos and Asian Americans are heterogeneous groups composed of both foreign- and US-born residents. Studies have pointed to further stratification of cancer incidence and mortality in these diverse populations based on birthplace.^77–81^ Data from California and Texas—states with a high proportion of Mexican Americans—showed higher mortality rates for Hispanics/Latinos compared with NHWs, with inverse rates between both states when comparing foreign- and US-born residents with NHWs.^81^ Because of the growing demographic impact of these two minority groups and the known high incidence of gastric cancer in AA/Bs, gastric cancer defines a leading cause of cancer health disparities in the USA.

### Potential aetiological factors

Differences in exposure to known risk factors for gastric cancer are likely to contribute to the observed disparities. For example, *H. pylori* infection has been associated with the development of non-cardia gastric cancer,^290,298,299^ and its prevalence is higher in racial/ethnic minority populations.^296^ Likewise, low-neighbourhood SES was found to be associated with an increased risk specifically for non-cardia gastric tumours.^296^ A separate study of SEER data in California found that most Hispanic/Latino and AA/B patients lived in lower SES neighbourhoods when compared with NHWs.^295^

### Gastric tumour subtypes

Hispanics/Latinos, AA/Bs, Asians and Hawaiian/Pacific Islanders are more likely than NHWs to suffer from non-cardia, diffuse-type gastric cancer,^290,291,296^ a histological subtype that is associated with treatment resistance and poor clinical outcomes, and that is the main driver of disparities between NHW and USA minority populations.^21^

Attempts have been made to further characterise gastric cancer at the molecular level. TCGA has identified four molecular subtypes of gastric cancer: Epstein–Barr virus (EBV)-associated, microsatellite instable (MSI), genomically stable (GS) and chromosomally instable (CIN).^304^ Importantly, these subtypes demonstrated differences in their therapeutic response: EBV is associated with a better prognosis, patients with a CIN subtype benefited most from adjuvant therapy and those with a GS subtype benefited least from adjuvant therapy and displayed worse prognosis.^305^ However, the vast majority of current genomic resources include samples from NHW individuals,^304^ and therefore little is known about the distribution of these subtypes in other populations. A whole-exome sequencing (WES) pilot study of 28 Latin American patients with gastric cancer found a lower prevalence of MSI (8% vs. 22%) and CIN (35% vs. 49%) subtypes, and a higher prevalence of EBV (14% vs. 8%) and GS (45% vs. 19%) compared with TCGA data, as well as significantly different frequencies of mutations in known driver genes (*ARID1A*, *PIK3CA* and *CDH1*) for gastric cancer between the two populations.^306^ Another study in Hispanics/Latinos from Texas also found that gastric tumours from these minority populations are enriched for the GS subtype.^307^ Together, these data suggest population differences in the aetiology and molecular subtype between NHWs and Hispanics/Latinos, many of which might have an effect on the prognosis and therapeutic response. Further identification of the distinct molecular mechanisms underlying the aetiology of gastric cancer in USA minorities will be critical for the development of effective treatments and preventive screening methods to address health disparities in this disease.

## Disparities in leukaemia

### Incidence and mortality

Leukaemia is a malignancy of haematopoietic tissue comprising four major subtypes: acute lymphoblastic leukaemia (ALL), acute myeloid leukaemia (AML), chronic lymphoblastic leukaemia (CLL) and chronic myeloid leukaemia (CML). Of these, ALL shows the most pronounced racial/ethnic disparity.^308–311^ For AML, CLL and CML, incidences are lower in non-NHW populations than in NHWs; however, AA/B patients have the lowest survival rates for these malignancies.^312^ For CLL, AA/B patients present at a younger age but at a more advanced stage, and have worse survival.^313,314^ Similarly, AA/B and Hispanic/Latino AML patients are diagnosed at a younger age, but with a higher frequency of favourable cytogenetic subtypes, although they have increased mortality compared with NHWs.^315–317^ Disparities are also observed among the less frequent haematologic malignancies, such as multiple myeloma, with a higher risk among AA/Bs than other racial/ethnic groups.^318,319^

ALL is the most common cancer in children, accounting for ~30% of paediatric malignancies.^320^ Children of Latin American origin have the highest risk of ALL in the USA, with age-adjusted incidence rates ~15–40% higher than for NHWs, and with some of the highest global incidences reported in Latin American countries, including Mexico and Costa Rica.^309–311,321–324^ The incidence of childhood ALL has been increasing in the past decades in the USA, and is rising fastest in Hispanics/Latinos, with an annual percent change significantly higher than in other racial/ethnic groups.^325^ Similarly, the incidence of adult ALL is the highest in Hispanics/Latinos.^326^ Moreover, outcomes are generally poorer for both childhood and adult ALL in Hispanics/Latinos compared with NHWs^309,327–331^ probably due to disparities in SES,^328,332^ an increased frequency of the high-risk Philadelphia chromosome (Ph)-like subtype^333–335^ and the increased Indigenous American genetic ancestry in Hispanic/Latino patients (see below).^17^

### Potential aetiological factors

Germline loss-of-function variants in *NUDT15*, which confer a major cause of treatment-related toxicity due to thiopurine intolerance, are more common in Hispanic/Latino ALL patients than in NHW patients—in particular, in Hispanics/Latinos with high Indigenous American ancestry.^336,337^ Increasing Indigenous American ancestry has also been shown to be associated with a greater risk of relapse in children with ALL.^17^ Genetic risk alleles in the *ARID5B* gene, which encodes an oncogenic factor involved in transcription, have been shown to be associated with increased Indigenous American ancestry, as well as an increased risk of relapse, supporting the notion that germline variation influences both ALL incidence and outcomes.^338^ Risk alleles in the additional ALL GWAS loci *CEBPE*, *GATA3* and *PIP4K2A* were also positively associated with Indigenous American ancestry.^339,340^ Furthermore, five established GWAS-identified SNPs for childhood ALL (*ARID5B*, *GATA3*, *PIP4K2A*, *ELK3* and 17q12) have a >10% higher risk allele frequency in Hispanics/Latinos than in Europeans in the Genome Aggregation Database^341^ compared with one locus (*LHPP*) that has a >10% higher frequency in Europeans. In addition, in the haematopoietic transcription factor gene *ERG*, a locus was identified in which SNPs conferred a stronger risk of ALL in Hispanics/Latinos than in NHWs, with effect sizes of ~1.6 and 1.1, respectively.^342,343^ Among Hispanics/Latinos, *ERG* risk alleles correlated positively with the extent of Indigenous American ancestry, and conferred a larger effect on ALL risk with increasing Indigenous American ancestry, both globally and at the haplotype level.^342,343^ Further research is required to determine whether the ethnicity-dependent effects of *ERG* might result from interaction with other genetic or non-genetic factors, and to discover additional ancestry-related risk loci via admixture mapping and larger GWAS of ALL in Hispanics/Latinos across all age groups.

Whereas the increased incidence of ALL in Hispanics/Latinos is thus likely to reflect a greater genetic susceptibility in this population, rising rates of ALL over a short period of time implicate environmental exposures. With the exception of ionising radiation,^344^ few environmental risk factors have been established for ALL. Exposure to tobacco smoke, pesticides, paint and other organic pollutants has shown a modest positive association with childhood ALL in Hispanics/Latinos and other racial/ethnic groups.^66–69,85,86^ Intriguingly, day-care attendance and higher birth order, proxies for early-life infectious exposure that support Greaves’ ‘delayed infection’ hypothesis,^345,346^ have been reported to confer protection in NHWs but not in Hispanic/Latino children.^347,348^ By contrast, however, both Caesarean section and in utero CMV infection conferred a larger risk of ALL risk in Hispanics/Latinos.^70,95^ Determining the mechanisms that underlie this heterogeneous response to immune-related risk factors, and examining these in conjunction with the increased burden of ALL risk alleles in Hispanics/Latinos (e.g. gene–environment interactions), will shed light on the aetiology of ALL.

## Disparities in liver cancer

### Incidence and mortality

Although the overall cancer death rate in the USA is declining for both men and women, the death rate for hepatocellular carcinoma (HCC) remained the fastest rising cause of cancer-related deaths from 1999 to 2013.^349^ The incidence rate of HCC also rose dramatically during this period, secondary only to that of thyroid cancer.^349^ It is two to three times higher in men than in women,^349^ with American Indian/Alaska natives having the highest rates, closely followed by Hispanics/Latinos and Asian/Pacific Islanders.^217^ Among Hispanics/Latinos, USA-born individuals were reported to have higher incidence rates than foreign-born.^350^ In California, Asians have a higher incidence of HCC than do NHWs, AA/Bs and Hispanics/Latinos, and within Asians, HCC is eight to nine times more common among Southeast Asians (Laotians, Vietnamese and Cambodians) compared with other Asian groups.^9^

### Potential aetiological factors

HCC occurs in the setting of chronic liver disease, and any aetiology of liver disease can increase the risk of HCC. However, the overwhelming cause of HCC worldwide is hepatitis B (HBV). In the USA, however, hepatitis C (HCV) has been the primary cause of the rise of HCC since the 1970s. Together, HBV and HCV infections account for 78% of cases of HCC in the USA.^351^ Alcoholic liver disease, non-alcoholic fatty liver disease (NAFLD) and non-alcoholic steatohepatitis (NASH) due to diabetes, obesity and dyslipidaemia, are other major causes.

HCC disparately affects disadvantaged and racial/ethnic minority populations,^73,352^ with wide geographic and racial/ethnic variations, which can be attributed to differential exposure to HBV and HCV, as well as disparate access to high-quality screening and preventive care. HBV is the most common cause of HCC among Asians and Hawaiian/Pacific Islanders.^353,354^ The rising prevalence of obesity and the metabolic syndrome, with the consequent increase in NAFLD, also contributes to the rising incidence of HCC in the USA. Hispanics/Latinos are disproportionately affected by NAFLD, with some studies estimating that this disease affects over 40% of USA Hispanic/Latino population.^355^

### Tumour biology

The most common somatic mutations in HCC include aberrations in the *TERT* promoter and the *CTNNB1* and *TP53* genes.^356^ Data from 373 HCC samples from the TCGA database showed that AA/Bs had the highest frequency (70%) of *TP53* mutations compared with Asians (36.5) and NWHs (22.8).^357^ There is no evidence of racial/ethnic differences in the frequency of CTNNB1 mutations.^358^ However, *CTNNB1*-activating mutations are associated with HCC arising in a background of alcoholic liver disease, and *TP53* mutations are commonly associated with HBV-induced HCC.^356^ Further studies are needed to explore how differential exposure to risk factors between racial/ethnic groups might affect the tumour mutational landscape.

### Healthcare, prevention and treatment

Despite advances in multimodality therapies for HCC, its prognosis remains relatively bleak compared with other cancers, with an estimated 5-year relative survival of 21%.^359^ Racial/ethnic disparities exist in the stage of disease at diagnosis as well as survival. A review of California Cancer Registry data between 1988 and 2012 showed that those individuals least likely to present with local (early) disease or undergo transplantation for HCC were Laotian/Hmongs, AA/Bs, AI/ANs and Filipinos.^359^ The same groups were also more likely to live in neighbourhoods with the lowest SES quintile, corroborating the idea that limited healthcare resources might contribute to later stage at diagnosis and lower rates of receipt of local/regional curative therapies. Furthermore, across the USA, AA/B and Hispanic/Latino men have the highest average person-years of life lost (21 and 20 years, respectively).^349^

Biomarker-selected therapy or trials for HCC remain limited, as it is difficult to specifically target known mutations. Therefore, in order to address the rising incidence of HCC and its associated morbidity, efforts must focus primarily on the prevention and control of HBV and on curative therapy for HCV as the primary underlying aetiologies of HCC. NAFLD poses a major concern for a rise in HCC in the near future, due to increasing disease prevalence and the absence of curative treatments. Efforts focusing on HBV, HCV and NAFLD prevention and treatment should prioritise populations that are most affected by economic, language or geography barriers.^360–362^

## Conclusions

Despite great progress in our understanding of factors that contribute to racial/ethnic disparities in cancer incidence, tumour biology and outcomes, disparities still exist, and multidisciplinary efforts are needed to ameliorate or eliminate them (Box 2).

Federal initiatives have promoted the accrual of diverse populations in research studies and clinical trials in the USA in order to increase our understanding of the potential variation in aetiology, tumour behaviour and treatment response.^363,364^ However, individuals from diverse racial/ethnic backgrounds still account for an extremely low percentage of participants.^30^ Additional efforts should be supported to systematise the detailed collection of data on biological (including the proportion of genetic ancestry), behavioural, physical/built environment, sociocultural environment and healthcare system factors so that we can further identify and understand the relevant levels of intervention that are required to reduce, and ultimately eliminate, cancer health disparities. Cell lines and patient-derived xenograft models should also be representative of racial/ethnic diversity to allow researchers to conduct experiments in genomic and cellular contexts that better embody human variation.

However, in order to eliminate cancer health disparities, increasing knowledge about its causes will not be enough. We need to address the lack of sufficient data to better understand cancer aetiology and develop appropriate treatments in diverse populations; also, it is of utmost importance to expand ongoing culturally and linguistically tailored programmes focused on cancer awareness, education and navigation, as well as programmess to promote behavioural changes in ‘at risk’ groups focused on already-known modifiable factors. Behavioural changes should also be supported by structural factors and policies that facilitate them, such as tobacco control. Finally, and most importantly, disparities will not be eliminated without the implementation of system changes that promote health equities, and universal health insurance coverage (with little or no co-pay) and access to high-quality care for all.

## Disclaimer

The content of this review is solely the responsibility of the authors and does not necessarily represent the official views of the National Institutes of Health or of any of the funding agencies.

## Data Availability

Not applicable.
